# Elevated Gamma Connectivity in Nidopallium Caudolaterale of Pigeons during Spatial Path Adjustment

**DOI:** 10.3390/ani12081019

**Published:** 2022-04-14

**Authors:** Mengmeng Li, Jiantao Fan, Lubo Lin, Zhigang Shang, Hong Wan

**Affiliations:** 1 Henan Key Laboratory of Brain Science and Brain-Computer Interface Technology, School of Electrical Engineering, Zhengzhou University, Zhengzhou 450001, China; mengmeng_li@gs.zzu.edu.cn (M.L.); 202012182013374@gs.zzu.edu.cn (J.F.); 2 Department of Biopsychology, Institute of Cognitive Neuroscience, Faculty of Psychology, Ruhr University Bochum, 44801 Bochum, Germany; 3 School of Intelligent Manufacturing, Huanghuai University, Zhumadian 463000, China; lubo-lin@stu.huanghuai.edu.cn; 4 Institute of Medical Engineering Technology and Data Mining, Zhengzhou University, Zhengzhou 450001, China

**Keywords:** spatial navigation, path adjustment, nidopallium caudolaterale, gamma, functional connectivity, pigeon

## Abstract

**Simple Summary:**

Imagine that you need to reach a designated destination, but the familiar path you most often choose suddenly becomes impassable. Then, what will you do? Of course, you will try to adjust the path according to your cognition of the current environment and the goal. During this, how will be the spatial environment, especially the path adjustment process, be represented in your brain? That is a very interesting research topic. In this study, we attempted to explore the internal neural patterns within the brain, especially within the higher-order cognitive brain areas, by taking pigeons, a species with excellent navigation ability, as a model animal. The most classical detour paradigm was used to train pigeons in a task of spatial path adjustment, and the neural signals in pigeons’ nidopallium caudolaterale ((NCL) functionally similar to mammalian “prefrontal cortex”) were recorded. We found that the spatial path adjustment process is accompanied by modifications of the changes in spectral and connectivity properties of the neural activities in the NCL. The elevated gamma connectivity in the NCL found in this study supports the role of the NCL in spatial cognition and contributes to explaining the potential neural mechanism of path adjustment.

**Abstract:**

Previous studies showed that spatial navigation depends on a local network including multiple brain regions with strong interactions. However, it is still not fully understood whether and how the neural patterns in avian nidopallium caudolaterale (NCL), which is suggested to play a key role in navigation as a higher cognitive structure, are modulated by the behaviors during spatial navigation, especially involved path adjustment needs. Hence, we examined neural activity in the NCL of pigeons and explored the local field potentials’ (LFPs) spectral and functional connectivity patterns in a goal-directed spatial cognitive task with the detour paradigm. We found the pigeons progressively learned to solve the path adjustment task when the learned path was blocked suddenly. Importantly, the behavioral changes during the adjustment were accompanied by the modifications in neural patterns in the NCL. Specifically, the spectral power in lower bands (1–4 Hz and 5–12 Hz) decreased as the pigeons were tested during the adjustment. Meanwhile, an elevated gamma (31–45 Hz and 55–80 Hz) connectivity in the NCL was also detected. These results and the partial least square discriminant analysis (PLS-DA) modeling analysis provide insights into the neural activities in the avian NCL during the spatial path adjustment, contributing to understanding the potential mechanism of avian spatial encoding. This study suggests the important role of the NCL in spatial learning, especially path adjustment in avian navigation.

## 1. Introduction

The detour paradigm is one of the oldest and most challenging paradigms in animal spatial cognition research [1]. In it, the familiar path will be blocked and a new non-preferred path must be found by the animal [2,3]. Previous studies showed that abundant spatial information will be ultimately projected to specific brain regions for processing and integration; the mechanism of spatial perception and behavior decision-making functions of the brain have been widely studied [4,5,6]. In the related experiments by Alvernhe et al. [7,8], rats shifted to the appropriate path very rapidly, if not immediately, in detour sessions after the preferred path was blocked. A large amount of research has shown that prefrontal cortex (PFC) is inextricably linked to complex, high-level cognitive functions and allow for flexible, adaptive, and goal-directed behavior, playing an important role in bridging the stimulus perception with the response execution in the distributed cortical network [9]. Further studies have shown that PFC plays an important role in spatial navigation [10] and may support the encoding and retrieval of the spatially related information [11,12]. For avian studies, the functional similar brain region, the nidopallium caudolaterale (NCL), was also reported to play an important role in the spatial cognition of birds [13,14].

As a comprehensive, convergent region in the avian brain, the NCL is considered to be an important integration center in the brain telencephalic pallium [15], which bridges the sensory input regions’ and the motor output regions’ functionally. The important role of the NCL can be confirmed by the numerous pathways between it and a large number of other regions [16]. The results of NCL damage experiments showed that the executive functions of birds such as reverse learning [17] and working memory [18] would be affected. In 2012, Güntürkün [19] described a case of convergence in the evolution of brain and cognition of mammals and birds, comparing the NCL to the avian “PFC”. Furthermore, in the review discussing the neural basis of birds’ navigation by Mouritsen et al. [20], the avian NCL was considered to play an important role in navigation-related behaviors including setting goals, selecting appropriate actions, and altering intermediate strategies when new and unexpected information becomes available. The experimental results also showed that the NCL supported real-time decision making in the goal-directed navigation behavior, in which the neural information reflected the choice of motion directions [21]. In our previous study, we found that the NCL cooperates with the hippocampus (Hp) during route formation to support the goal-directed spatial learning of the pigeons [22]. While these studies reviewed the role of the avian NCL in spatial cognition and explored the neural patterns in spatial navigation behaviors, we still only have limited knowledge about the specific immediate neural response when animals encountered the sudden change of spatial environment and a sudden detour was needed to find the learned goal, for example.

Thus, we attempted to explore the neural patterns within the brain, especially within the higher-order cognitive brain areas, by taking Rock Pigeon (*Columba livia*), an amazing species with excellent navigation ability, as a model animal. We designed a goal-directed spatial task with the detour paradigm for pigeons and recorded the behavioral and neural data during the experiment. In the task, we trained the pigeons to learn a preferred path to the goal in the maze, firstly, and then we blocked the learned path. The pigeons had to learn to adapt to the sudden changes of the environment and find a new, alternative path to get the food reward in the goal. Moreover, we intended to understand how the neural patterns in the NCL are correlated with the behavior during the whole spatial cognitive task. We predicted that the pigeons would progressively learn to solve the path adjustment task when a forced detour was required to find the goal. Furthermore, there may be specific neural pattern changes in the NCL between different phases.

## 2. Materials and Methods

### 2.1. Subjects, Surgery, and Electrode Implantation

Six pigeons weighing 400 to 500 g were used in our current experiment, numbered as P090, P094, P097, P100, P110, and P130, respectively. All of the experiments were conducted according to the Animals Act, 2006 (China), for the care and use of laboratory animals and approved by the Life Science Ethical Review Committee of Zhengzhou University.

**Figure 1:**
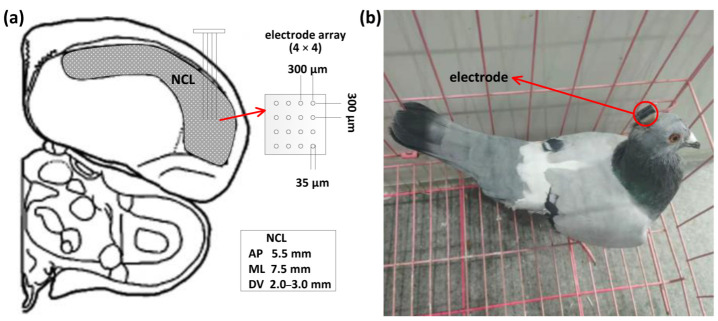
Implanting locations, microelectrode arrays, and a pigeon with implanted electrodes. (**a**) Diagram of the implanting location and microelectrode array. AP: anteroposterior; ML: mediolateral; DV: dorsoventral. (**b**) A pigeon (pigeon ID: P097) with implanted electrodes’ matrix.

All pigeons were housed in an animal facility for at least 2 weeks prior to the surgeries. At the beginning of the surgery, the pigeon was anesthetized with 1.5% pelltobarbitalum natricum (0.25 mL/100 g) injected intraperitoneally. Then, we placed the pigeon in a stereotaxic apparatus and the brain operation was performed, in which we chronically implanted a recording microelectrode array (4 × 4 = 16 channels; platinum iridium alloy; tip spacing. 300 μm; diameter, 35 μm; impedance, 0.5–1.0 MΩ; Hong Kong Plexon Inc., Hong Kong, China) in the left NCL (anteroposterior: 5.5 mm; mediolateral: 7.5 mm; dorsoventral: 2.0 to 3.0 mm). The localization of the NCL was carried out according to the Karten and Hodos stereotaxic atlas of the pigeon brain [23]. After the recovery period, a free-foraging task in a circular maze (diameter, 1 m; height, 80 cm) was used to test the cognitive ability of the subjects. No side effects were observed during and after the surgeries of all pigeons. We show the implanting location, the microelectrode array (Figure 1a), and a pigeon with the implanted electrode as the example (Figure 1b).

### 2.2. Spatial Cognitive Experiment and Apparatus

**Figure 2:**
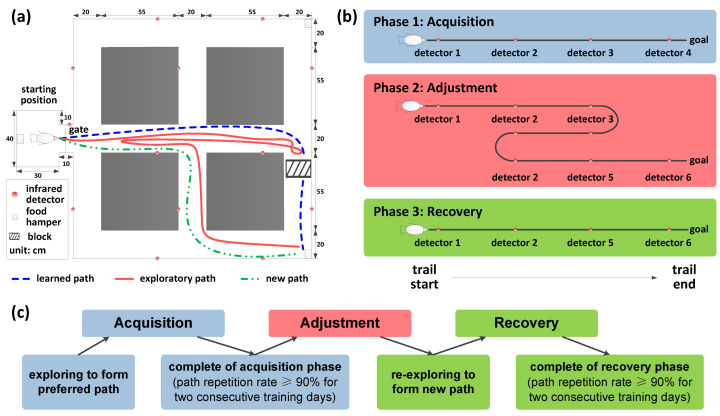
The apparatus and procedures (one of the sessions as an example). (**a**) Diagram of the maze apparatus for pigeons and the goal-directed spatial task. The pigeon was trained to learn a preferred path to the goal firstly. After the acquisition, this path was blocked and the pigeon had to adjust the path to access the goal, exploring in the maze to find a new path. Finally, it learned a new path, recovering from the adjustment. (**b**) The acquisition, adjustment, and recovery phases of the experiment. (**c**) Experimental task procedures visualizing the learning of the pigeon.

The pigeons implanted with electrode arrays were taken to our designed maze (Figure 2a) to begin the experiment after the recovery period of about 1 week. There was a starting position as the waiting area and two alternative positions as the goals in the maze. In these three above positions, food hampers were set to provide food rewards. The infrared detectors distributed along the paths were used to define the beginning and end time of animal displacements in the maze for signal segmentation.

According to our three-phase, goal-directed, spatial cognitive task procedure (Figure 2b), the animal was trained to explore the maze and find the goal with the food hamper through a path at the first phase. When the pigeon could reliably perform the task through a preferred path, in which the path repetition rate reached more than 90% of the total trial numbers for 2 consecutive days, it was considered that Phase 1 (acquisition) of the experiment was completed. Then, the experiment went into Phase 2 (adjustment), in which the learned preferred path was blocked at the pathlet close to the goal. In this phase, the pigeon had to reexplore the maze to find a new path to the goal, dealing with the sudden change of the spatial environment. Generally, the pigeons learned to solve the path adjustment task after multiple trials and the experiment entered Phase 3 (recovery). Finally, the pigeons were used to accessing the goal through the new path and adapted to the changes in the spatial environment after path adjustment.

For each pigeon, the experimental process including the above three phases was considered as a session, in which one of the two alternative goal positions was chosen as the goal randomly. Each experimental phase consisted of a number of trials; in each trial of different phases, the food hamper at the goal was opened firstly to provide food rewards. After the pigeons found the goal and enjoyed the food for 5 s, the food hamper at the goal was closed and the hamper at the starting position was opened. Finally, the pigeon had to go back to the starting position to get the food and wait for the next trial.

### 2.3. Behavioural Data and LFPs’ Recording

The behavioral data of the pigeons performing the spatial task were recorded by the observation camera placed on the ceiling. We analyzed the trajectories and timings of the pigeons to calculate two behavioral indicators of different cognitive phases, the average time the pigeons spent per session and the path length the pigeons walked from the starting position to the goal. Generally, it took several days to complete the training of a whole session in our experiment. First, the pigeons needed 3.94 ± 0.77 (3–5) days to learn a stable preferred path. Then, in the path adjustment phase, it took the pigeons 1.19 ± 0.40 (1–2) days to get familiar with the changes of the maze environment and adjust their navigation strategy to overcome the dependence on the learned path. Finally, a new, stable path was formed again, which took about 3.38 ± 0.50 (3–5) days for the pigeons. For all sessions, the average time the pigeons spent per session and the path length the pigeons walked from the starting position to the goal under different experimental phases (acquisition, 525 trials; adjustment, 133 trials; and recovery, 620 trials) were calculated.

Our neural recording system was a 128-channel Cerebus^TM^ Multichannel Acquisition Processor (Blackrock Microsystems, Salt Lake City, UT, USA). LFPs from the NCL region of the pigeons were recorded at the sampling rate of 2 kHz using an analog 16-channel Samtecs headstage (20 pins, Blackrock Microsystems, Salt Lake City, UT, USA). The signals were transmitted through the connecting wires and amplified at the amplification gain of 300 by a Cerebus processor. A 0–250-Hz Butterworth low-pass filter was used for signal filtering. Bad channels caused by detached electrode contacts, intermittent electrical connection, or line noise were removed from all 16 channels [24]. The signals of interest (SOIs) corresponding to different cognitive states from the three phases of the experiments were segmented. Finally, signals at five different sub-bands, including delta (1–4 Hz), theta (5–12 Hz), beta (13–30 Hz), slow-gamma (31–45 Hz), and fast-gamma (55–80 Hz), for each SOI segment were filtered and obtained for further analysis.

### 2.4. Spectral Analysis

Power spectral density (PSD) can be used to analyze the signal and its intensity from the perspective of the frequency domain [25]. In this paper, we used the autoregression (AR) model power spectrum estimation to calculate the energy density in each frequency band.

### 2.5. Functional Connectivity Analysis

The functional connectivity properties in the NCL of the pigeons during the experiment were used for the spatial cognitive functional representation. We defined the channels of the electrode array as the nodes of the functional network, and the connections between the channels as the edges. The coherence coefficient [26] of the LFPs corresponding to the channels was used to construct the functional network based on graph theory. For a given frequency f, the coherence between two LFP time series, x and y, was calculated as follows:

(1) $$Coh_{x,y}(f)=\frac{{\left|p_{x,y}(f)\right|}^{2}}{\left|p_{x}(f)\right|\times \left|p_{y}(f)\right|},$$

where

(2) $$p_{x,y}(f)=\frac{1}{n}\sum_{i=1}^{n}x_{i}(f)y_{i}^{*}(f),$$

For a given frequency f, $p_{x}(f)$ and $p_{y}(f)$ represent the auto-power spectrums of two LFP time series x and y, respectively, and $p_{x,y}(f)$ is the cross-power spectrum.

To explore the functional connectivity in the NCL during different phases (acquisition, adjustment, and recovery) in this paper, we calculated the coherence coefficients between the LFPs of different channels in the NCL and visualized their corresponding matrices’ heat maps in different bands. Then, we binarized these matrices to visualize the network connections more concisely and clearly. The coefficient value in the heat map represents the connection strength between any two channels, measuring the functional relationship between them. The coefficient value is represented by the color; as it increased, the color appeared to turn yellow and, conversely, turn blue. Then, the appropriate threshold was selected for the binarization of each matrix, in which the value below the threshold in the matrix was set to 0, and the others above the threshold were set to 1 [27]. Finally, the thresholded new matrix was used for network visualization, in which the edge corresponding to 0 would be removed and the edge corresponding to 1 remained. For further quantitative analysis, the fitted normal density curves estimated from the data of the coherence coefficient matrices of different phases in all bands were obtained and the expected values were compared quantitatively.

The statistical measures of the topological characteristics are important for the quantitative analysis of the functional connectivity for the network [28,29]. To further analyze the network connection patterns of different phases quantitatively and test whether the functional connectivity characteristics showed specific modifications along with the changes of the phases in the task, the topological properties of the network including the clustering coefficient (*Coef*) and global efficiency (*Eff*) were calculated from the perspective of graph theory.

The clustering coefficient, a local characteristic of the network measuring the degree to which the nodes tend to gather, reflects the connectivity intensity of the network, which is calculated as follows:

(3) $$coef=\frac{1}{M}\sum_{i=1}^{M}\frac{2E_{i}}{k_{i}\left(k_{i}-1\right)},$$

where $k_{i}$ indicates that the i-th node has k connections with the other channels, $E_{i}$ indicates the number of connections in the network that connect with the i-th channel, and M is the total number of channels in the network. The value of the clustering coefficient ranges from 0 to 1. The larger the clustering coefficient is, the higher the modularity of the network is.

Global efficiency, a global characteristic of the network, reflects the information transmission performance of the network, which can be defined as:

(4) $$Eff=\frac{1}{M}\sum_{i\in M}^{}\frac{\sum_{j\in M}{\left(d_{ij}\right)}^{-1}}{M-1},$$

where $d_{ij}$ indicates the shortest path length between two channels, i and j. The value of global efficiency ranges from 0 to 1. The larger the global efficiency is, the stronger the information transmission capacity of the network is.

### 2.6. PLS-DA Multivariate Model

Partial least square discriminant analysis (PLS-DA) [30] was used to extract meaningful functional connectivity features that distinguish different phases of acquisition, adjustment, and recovery and visualize the trial groups from them. As a typical supervised discriminant analysis method for multivariate data analysis, PLS-DA is often used to deal with classification and discrimination problems. PLS-DA performs projection analysis on the raw data structure, firstly, based on partial least squares method, searching the latent variables represented as the linear combinations of the original variables. Then, the latent variables with the largest covariance with the original variables are determined and used in the modeling analysis to find the sources of the data variability. Latent variable scores and loadings provide a graphical visualization and understanding of the different data patterns and relations. Scores represent the coordinates of the samples in the projected space, while loadings are the coefficients of variables in the linear combinations, which can be interpreted as the influence of each original variable on each latent variable [31]. PLS-DA combines the advantages of partial least squares regression and supervised classification technology. It can effectively distinguish the observed variables between groups and further determine the variables leading to the differences between groups. Therefore, we here applied PLS-DA for multivariate cognitive process modeling based on the functional connectivity features, providing separability discriminant features and corresponding component analysis for different phases during the spatial task, further verifying the robustness of the specific concomitant change of the internal functional connection features in the NCL along with the behavior during path adjustment. The toolbox we used was the classification toolbox for MATLAB (https://michem.unimib.it/download/matlab-toolboxes/classification-toolbox-for-matlab/ (accessed on 6 November 2021)) from University of Milano-Bicocca.

We built a PLS-DA multivariate model in this paper to provide adequate separation of the three different phases from the task and robust analysis of connectivity variables. The multivariate data set included all trial samples from three phases during acquisition, adjustment, and recovery, described by 10 variables. These variables were topological features of functional connectivity belonging to five frequency bands, including delta_c, delta_e, theta_c, theta_e, beta_c, beta_e, slow-gamma_c, slow-gamma_e, fast-gamma_c, and fast-gamma_e, where “_c” represents the clustering coefficient feature while “_e” represents the global efficiency feature.

### 2.7. Statistical Analysis

All statistical analyses were performed by MATLAB R2014a software (The MathWorks, Inc., Natick, MA, USA), in which the results were presented as mean ± standard deviation (std). The statistically significant difference level of the used rank-sum test was set to 5% with the statistically significant differences indicated by a *p* value as follows: * *p* < 0.05, ** *p* < 0.01, *** *p* < 0.001. A post hoc test using Dunn’s test with a Bonferroni correction was used and epsilon square *ε*^2^ was computed as an estimate of the effect size [32].

## 3. Results

### 3.1. Behavioural Results

**Figure 3:**
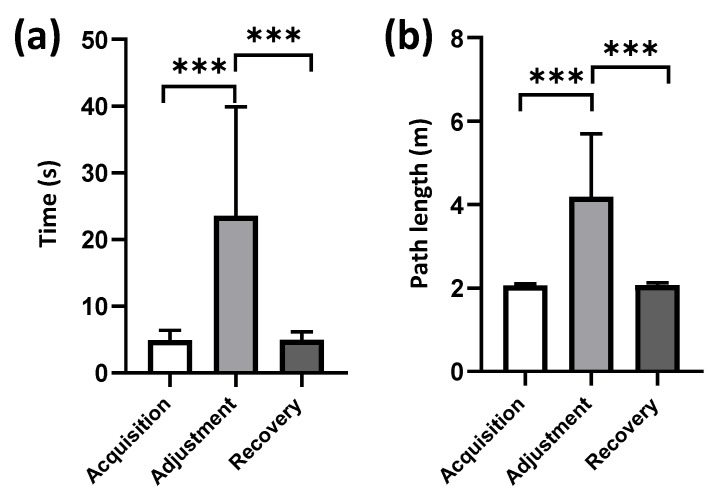
Average time spent and path length walked by pigeons across different sessions. Error bars indicate standard error (number of pigeons: 6; total trials for acquisition, adjustment, and recovery: 525, 133, 620, respectively). Significant differences are indicated by star marks (*** *p* < 0.001). (**a**) Average time. (**b**) Average path length.

The average times for acquisition, adjustment, and recovery phases were 4.93 ± 1.44 (2.04–12.53) s, 23.54 ± 16.31 (6.25–82.69) s, and 4.98 ± 1.18 (2.04–10.60) s (Figure 3a). The average path lengths for three phases were 2.06 ± 0.04 (2.00–2.19) m, 4.18 ± 1.51 (2.45–11.10) m, and 2.07 ± 0.06 (2.00–2.67) m (Figure 3b). The pigeons took a significantly longer time to find the goal during the adjustment phase than during the phases of acquisition (*x*^2^ = 305.89, *ε*^2^ = 0.47, *p* < 0.001, rank-sum test; Figure 3a) and recovery (*x*^2^ = 320.31, *ε*^2^ = 0.43, *p* < 0.001, rank-sum test; Figure 3a), while the durations of the phases of acquisition and recovery showed no significant difference. The path lengths of the adjustment phase were significantly longer than the other two phases (adjustment vs. acquisition: *x*^2^ = 321.68, *ε*^2^ = 0.49, *p* < 0.001; adjustment vs. recovery: *x*^2^ = 331.51, *ε*^2^ = 0.44, *p* < 0.001, rank-sum test; Figure 3b), while the path lengths of the phases of acquisition and recovery showed no significant difference.

### 3.2. Neural Results

**Figure 4:**
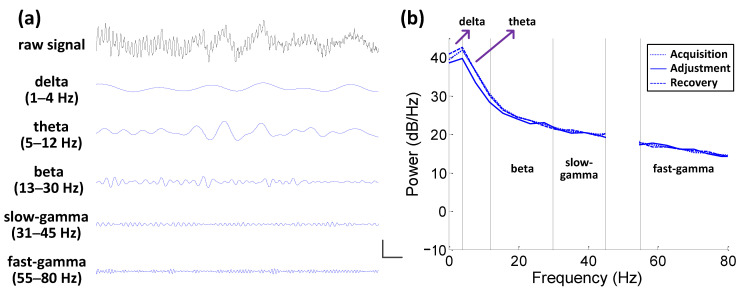
Spectral analysis of the neural activity during the spatial task. (**a**) Examples of simultaneous local field potentials recorded from the nidopallium caudolaterale and filtered at delta, theta, beta, slow-gamma, and fast-gamma frequency bands (calibration bar: 500 µV, 500 ms; from the pigeon numbered P097). (**b**) Mean power spectrum curves of three phases (number of pigeons: 6; trials for acquisition, adjustment, and recovery: 525, 133, 620, respectively) corresponding to different bands (delta, 1–4 Hz; theta, 5–12 Hz; beta, 13–30 Hz; slow-gamma, 31–45 Hz; fast-gamma, 55–80 Hz).

We implanted a 16-channel microelectrode array in the NCL of each pigeon. After bad-channel removal, we finally obtained 12, 15, 13, 12, 10, and 12 effective channels for subjects P090, P094, P097, P100, P110, and P130, respectively. We obtained simultaneous LFPs recorded from the NCL and the filtered signals at different bands (Figure 4a).

#### 3.2.1. PSD Analysis

Based on the comparisons of the mean PSD curves during different phases, it was seen that, in the lower-frequency bands (including delta, theta, and beta), the PSD during the phase of adjustment was slightly lower than that at the other two phases (Figure 4b). Furthermore, this kind of decreasing trend was less and less obvious with the increase in frequency. Finally, in the higher-frequency bands (including slow-gamma and fast-gamma), there was no obvious difference in the PSD curves among the different three phases (Figure 4b). The results showed that there was an internal correlation between the power spectral characteristic of the LFP signals in the lower-frequency band of the NCL and the spatial path adjustment.

#### 3.2.2. Functional Connectivity Analysis

**Figure 5:**
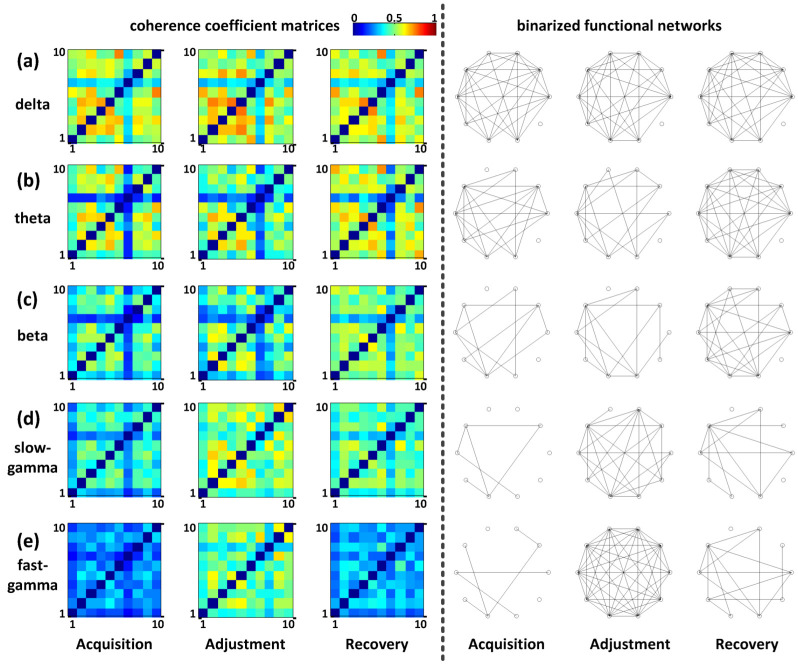
The heat maps of the coherence coefficient matrices and binarized functional networks for different bands in NCL; (**a**) delta; (**b**) theta; (**c**) beta; (**d**) slow-gamma; (**e**) fast-gamma. In the heat maps, the rows and columns of the matrices indicate the channel indexes of NCL. The coefficient value is represented by the color; as it increased, the color appeared to turn yellow and, conversely, turn blue. In the networks, each hollow circle indicates a channel, and the fewer connections between channels, the sparser the connections of the functional networks.

Taking P110 as an example, it was seen intuitively that there was no significant difference between the coherence matrix heat maps of all low-frequency bands including delta, theta, and beta (left, Figure 5a–c). Their corresponding functional network connection patterns at three different spatial cognitive phases were also similar (right, Figure 5a–c). However, in gamma bands, we found obvious differences in the results between the different phases (Figure 5d,e). For the heat maps, we found more blue pixels in the phases of acquisition and recovery than during the adjustment phase, especially in the fast-gamma band (left, Figure 5d,e). Furthermore, the connections during the adjustment were obviously more than in the other two phases (Right, Figure 5d,e). These results indicated that the spatial path adjustment elevated the functional connectivity of the NCL in the slow-gamma and fast-gamma bands.

**Figure 6:**
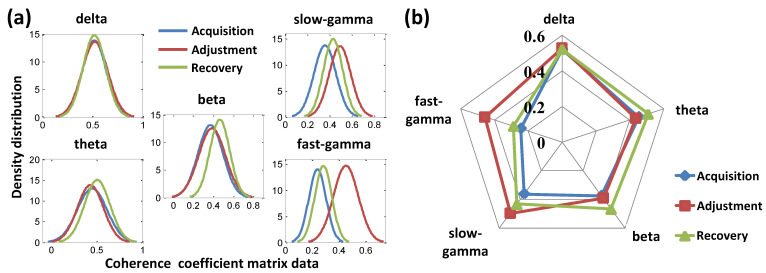
Fitted normal density distribution curves of the coherence coefficient matrices data and radar map of the expected values for different bands. (**a**) Fitted normal density distribution curves. (**b**) Radar map of the expected values.

The fitted density curves corresponding to the adjustment phase did not show an obvious numerical shift in the delta, theta, and beta bands but showed the trend of a larger data value shift in the two gamma bands compared with the other two phases (Figure 6a). The further quantitative description results based on the expected value radar map of the above curves showed that the expected values of the gamma bands during the adjustment phase were relatively higher than the other phases (Figure 6b).

**Figure 7:**
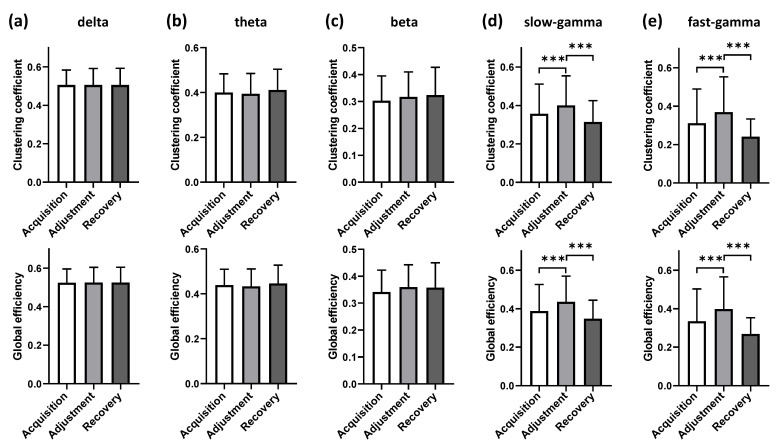
Comparative analysis of network topological properties during different phases for all sessions in five bands. Significant differences are indicated by star marks (*** *p* < 0.001); (**a**) delta; (**b**) theta; (**c**) beta; (**d**) slow-gamma; (**e**) fast-gamma.

The internal functional connectivity patterns of the NCL during the spatial path adjustment showed frequency band-specific changes compared with the other two phases (Figure 7). Specifically, there were no significant differences in the two functional connectivity characteristics for the low-frequency bands (delta, theta, and beta) among the different phases (Figure 7a–c), while those for the high-frequency bands (slow-gamma and fast-gamma) during adjustment were significantly higher than during acquisition (slow-gamma: *x*^2^ = 35.08, *ε*^2^ = 0.02, *p* < 0.001 for clustering coefficient and *x*^2^ = 60.10, *ε*^2^ = 0.04, *p* < 0.001 for global efficiency; fast-gamma: *x*^2^ = 47.03, *ε*^2^ = 0.03, *p* < 0.001 for clustering coefficient and *x*^2^ = 68.25, *ε*^2^ = 0.05, *p* < 0.001 for global efficiency, rank-sum test; Figure 7d,e) and recovery (slow-gamma: *x*^2^ = 107.38, *ε*^2^ = 0.07, *p* < 0.001 for clustering coefficient and *x*^2^ = 159.98, *ε*^2^ = 0.11, *p* < 0.001 for global efficiency; fast-gamma: *x*^2^ = 172.38, *ε*^2^ = 0.12, *p* < 0.001 for clustering coefficient and *x*^2^ = 225.89, *ε*^2^ = 0.15, *p* < 0.001 for global efficiency, rank-sum test; Figure 7d,e).

As stated above, we detected no obvious difference specifically in the gamma power of the LFP activity in the NCL (Figure 4b). All the associations between the spectral power and the spatial task phase were detected in the other bands. Moreover, PSD in these lower bands showed decreasing trends during adjustment (Figure 4b), in contrast to the increasing trend detected in the NCL connectivity (Figure 7d,e).

#### 3.2.3. Validation of Spatial-Associated Functional Connectivity Signatures

**Figure 8:**
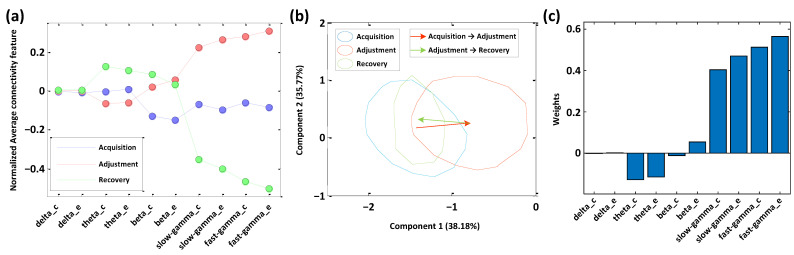
Multivariate PLS-DA modeling analysis of the three phases, acquisition to recovery. (**a**) The normalized average connectivity feature distribution of the three phases. (**b**) The projected distribution of all samples on the model’s first and second principal components. (**c**) The loading weights of each original feature on the latent variables corresponding to the first component.

The normalized average network connectivity feature distributions corresponding to the three phases of the task indicated that the obvious connectivity differences in the results among the different phases were observed at the high-frequency bands (slow-gamma and fast-gamma), while we found no distinct difference at the low-frequency bands (Figure 8a).

The multivariate PLS-DA model describing the spatial path adjustment process and the latent variable scores of the original variables of the samples from different phases projected to the first two latent components indicated that the first component explained 38.18% of the variance of the original variables, containing most of the original information (Figure 8b). Even though the distributions of the three phases overlapped to a certain extent, the scores of the samples during the path adjustment phase on the first component were higher than the other two phases (Figure 8b), while the score distributions of all samples on the second component showed no obvious differences (Figure 8b). Notably, the mentioned differences were mainly reflected between the path adjustment phase and the non-adjustment phases (including the acquisition and recovery phases). For the comparison between the acquisition and recovery phases, there was no significant difference found from the results of the modeling analysis (Figure 8b).

On the model’s first component, the weights of the connectivity features indicated that the representation contribution of the spatial phases’ difference basically came from the gamma bands (slow-gamma and fast-gamma), while the influences of the other bands were minimal (Figure 8b). These results are in line with the conclusion of the specific gamma connectivity during the path adjustment in the above topological analysis.

## 4. Discussion

In this study, we explored the behavioral and neural responses of the pigeons during a goal-directed spatial task. We found, facing the immediate detour situation, the pigeons progressively learned to solve the path adjustment task, as we predicted. Our results showed that the spatial path adjustment was associated with the changes in the spectral and connectivity properties of the NCL activities. From the perspective of behavioral data analysis, the results indicated that two behavioral indicators, the average durations and the path lengths the pigeons walked from the starting position to the goal during the spatial path adjustment phase, were both significantly longer than those during acquisition and recovery. From the perspective of neural data analysis, we found that spatial path adjustment in goal-directed behavior was associated with the changing of the PSD power properties in the lower bands of a pigeon’s NCL. Importantly, we found elevated functional connectivity in the gamma bands in the NCL, which increased significantly during adjustment than during the other phases. Furthermore, the PLS-DA modeling provided a more specific and robust basis for the connectivity feature analysis and identification of different phases in the process of path adjustment.

Increasing gamma connectivity in the NCL may correlate with the changes of the oscillatory activity amplitude. Therefore, we tested whether the NCL connectivity during adjustment was modulated by the PSD characteristics of the NCL. Our results suggested that the elevated NCL gamma connectivity during spatial path adjustment was not dependent on the increase in spectral power; it was more likely the result of the shared response patterns of the population of neurons in NCL.

The band-specific connectivity changes in gamma during the spatial path adjustment suggested a possible role of the gamma oscillations of the NCL in spatial learning and the adjustment of navigational strategy. Still, the correlations between the neural patterns and the performance of more specific behavioral patterns, governed by higher cognitive functions, such as working memory, should be further studied. Mammalian studies proved that the PFC neurons contribute the leading role in working memory [33,34] and the maintenance of working memory is mainly reflected in the continuous gamma oscillation [35,36,37]. Similarly, the results of avian studies also suggest an important role of the gamma rhythm from the NCL in spatial goal-directed behaviors. The time–frequency [38] and functional network characteristics [39] of LFP signals in the gamma band recorded from the NCL of pigeons, which is thought to be the similar region to the mammalian PFC, both showed correlations with the behavioral performance. The elevated gamma connectivity in the NCL in this study was consistent with the previous studies mentioned above. In this context, our results support the hypothesis that increased gamma activities are related to spatial working memory [40,41]. We suggest that this kind of enhancement may correlate with pigeons’ cognitive function organizing need to overcome the decision dilemma during path adjustment.

Our findings showed the correlation between the neural patterns in the NCL and the behavioral patterns during path adjustment, suggesting the modulation relationship between these two kinds of patterns. However, the more specific participation patterns of the NCL in path adjustment and the detailed causality between the neural and behavioral patterns still need more in-depth research, considering the role of the NCL as a converging brain area and its key role in adaptive functions including spatial navigation. Furthermore, just one, single brain region is not enough to be responsible for the complex spatial processing alone. A previous study proved that spatial navigation relied on a network of strongly interconnected structures including the PFC (showing similarities at a functional level with the avian NCL), Hp, and the basal ganglia [42]. Early researchers advocated the hypothesis that Hp is involved in the formation of new memories by acting as an intermediate-term buffer store for information about episodes, particularly for spatial information [43]. Studies in rodents also indicated that Hp encoded specific environments by place cells acting cooperatively, performing both pattern completion and pattern separation [44]. Additionally, hippocampal place cell remapping across the various tasks has been characterized as reflecting the coding of distinct memories within the same environment [45]. For avian Hp, our previous study also showed correlations between the behavioral patterns and specific hippocampal neural patterns during the spatial path adjustment of pigeons [46]. Specifically, the depressed connectivity in the lower bands (delta and theta) and elevated connectivity in the higher bands (gamma) in pigeons’ Hp were observed. All these above studies provide us positive guides and inspiration to continue the current study in this paper.

The different contributions of the neocortex and Hp in learning and memory has attracted much attention. A set of principles has been derived from converging biological, psychological, and computational constraints, including the neocortex’s extracting the general statistical structure of the environment using a slow learning rate and overlapping distributed representations, the Hp’s encoding the details of specific events using separated representations rapidly, error-driven and Hebbian nature of learning, and recall of information via pattern completion [47]. For birds, although the interaction studies focusing on the relationship between Hp and the NCL are still inconclusive, some studies have shown that these two regions both contribute cooperatively to the goal-directed, spatial-related decision-making tasks [48]. Recently, a study recording the neural signals in the Hp and the NCL of pigeons performing goal-directed, decision-making tasks suggested the existence of a causal functional information flow between them [49]. Therefore, we have reasons to believe that these two regions may also interact with each other in spatial learning, especially involved in the case of the path adjustment needs. The coupling of specific neural oscillations can be regarded as the bridge of the communications between regions. Early researchers believed that gamma oscillations with high-frequency characteristics may be used as spatial-related information inputs, which flow between different brain regions, relying on the loading of the other frequency bands [50]. Another study based on a spatial goal-directed behavior experiment of pigeons explored the information interaction mechanism between the Hp and NCL based on gamma oscillations, and the results showed that the coupling of the gamma LFP signals from the Hp and NCL was significantly higher than that in other bands [49]. These results provide supports for further research on the neural information patterns in the Hp–NCL local network modulated by spatial path adjustment.

On the other hand, as an avian brain region involved in a series of executive functions including decision making and high-order multimodal information processing [51], the NCL is also considered to support these processes based on different neural rhythms from the Hp [52]. Although their gamma consistency implies the sharing of a neural information pattern between them to a certain extent, there are still differences between the results of other frequency bands. Previous studies showed that the coordination of neural oscillations in different frequency bands features the comprehensive function of the brain [53,54,55]. For example, the nesting of fast and slow oscillations is considered to be conducive to the cross-modal interactions between the multisensory areas [56]. Therefore, cross-frequency coupling in the Hp–NCL local network is also an important direction for further research, focusing on how the neural oscillations of different rhythms interact with each other during the spatial cognitive process and the representation of the spatial path adjustment by the coordinated activities of multiple bands.

Overall, we showed that behavioral changes during spatial path adjustment are accompanied by modifications in functional connectivity in the NCL of pigeons, as well as changes in the oscillatory power. Altogether, these results provide insight into the dynamics of the neural patterns in the avian NCL during path adjustment in the goal-directed spatial cognitive task, contributing to revealing the potential mechanism of path adjustment in spatial learning.

## Data Availability

The datasets analyzed in the current study are available from the corresponding author on reasonable request.
